# The Platelet–Virus Axis in Human Disease

**DOI:** 10.3390/v18020183

**Published:** 2026-01-29

**Authors:** Carmine Siniscalchi, Manuela Basaglia, Egidio Imbalzano, Pierpaolo Di Micco

**Affiliations:** 1 Department of Internal Medicine, Parma University Hospital, 43100 Parma, Italy; csiniscalchi84@gmail.com (C.S.); mbasaglia80@gmail.com (M.B.); 2 Department of Clinical and Experimental Medicine, University of Messina, 98121 Messina, Italy; egidio.imbalzano@unime.it; 3 Internal Medicine Ward, P.O. Pozzuoli, ASL Napoli2 Nord, 80078 Pozzuoli, Italy

**Keywords:** platelets, viral infections, immuno-thrombosis, extracellular vesicles, thrombocytopenia, hypercoagulability, endothelial dysfunction, innate immunity, platelet activation, platelet–leukocyte interactions

## Abstract

Platelets have traditionally been viewed as passive cellular elements involved in hemostasis and vascular integrity. However, growing evidence over the last decade has radically changed this paradigm, revealing platelets as dynamic immune and inflammatory effectors that actively participate in host–pathogen interactions. In viral infections, platelets are not merely innocent bystanders but represent key players in a bidirectional and tightly regulated platelet–virus axis that influences viral dissemination, immune activation, endothelial dysfunction, and the development of thrombotic and hemorrhagic complications. Several clinically relevant viruses, including SARS-CoV-2, influenza virus, HIV, dengue virus, and viral hemorrhagic fever-associated pathogens, have been shown to directly or indirectly interact with platelets through surface receptors, immune complexes, and inflammatory mediators, leading to platelet activation, phenotypic reprogramming, and accelerated clearance. These processes contribute to the paradoxical coexistence of thrombocytopenia and hypercoagulability that characterizes many severe viral diseases. Moreover, platelets can act as immune sentinels by sensing viral components, releasing cytokines and chemokines, forming platelet–leukocyte aggregates, and modulating both innate and adaptive immune responses, thereby shaping the clinical course of infection. In this review, we synthesize current evidence on the molecular and cellular mechanisms governing virus–platelet interactions, with particular emphasis on their role in immune-thrombosis, endothelial injury, and organ dysfunction. We further discuss the clinical implications of platelet dysregulation in viral infections, including its potential value as a biomarker of disease severity and as a therapeutic target. Understanding the platelet–virus axis provides a unifying framework to explain the thrombo-inflammatory phenotype of viral diseases and may open new avenues for risk stratification and targeted interventions in affected patients.

## 1. Introduction

**Figure 1:**
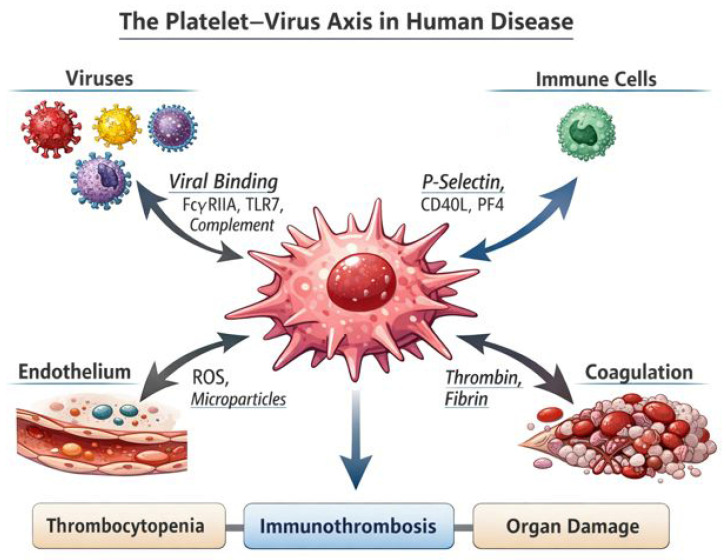
The platelet–virus axis in human disease. This schematic illustrates the central role of platelets as integrators of viral sensing, immune activation, endothelial dysfunction, and coagulation. Viral particles and virus-containing immune complexes interact with platelets through surface receptors such as FcγRIIA, Toll-like receptor 7 (TLR7), and complement receptors, leading to platelet activation and phenotypic reprogramming. Activated platelets release inflammatory and immune mediators, including P-selectin, CD40L, and platelet factor 4 (PF4), which promote platelet–leukocyte interactions and amplify innate and adaptive immune responses. In parallel, platelets contribute to endothelial injury through the release of reactive oxygen species (ROS) and microparticles, while enhancing thrombin generation and fibrin formation within the coagulation cascade. These interconnected pathways converge on the development of immune-thrombosis, platelet consumption, and tissue hypoxia, ultimately resulting in thrombocytopenia, microvascular thrombosis, and organ damage. The figure highlights how platelets act as key hubs linking viral infection to thrombo-inflammatory pathology in human disease.

Platelets have long been regarded primarily as key effectors of haemostasias and vascular integrity. However, growing evidence has deeply revised this concept, identifying platelets as active sentinels of innate immunity capable of sensing danger signals, interacting with leukocytes and endothelial cells, and orchestrating thrombo-inflammatory responses during infectious diseases [1]. In viral infections, platelet alterations are particularly prominent: many viral diseases are characterized by thrombocytopenia and/or platelet dysfunction, yet paradoxically display a high burden of thrombotic complications, both at the macrovascular and microvascular level [2,3]. This apparent contradiction highlights the central role of the platelet–virus axis in shaping disease severity, organ dysfunction, and clinical outcomes. Collectively, these data support a unifying model in which platelets act as central hubs integrating viral sensing, immune activation, endothelial dysfunction, and coagulation, thereby driving the thrombo-inflammatory phenotype of viral diseases (Figure 1).

From a mechanistic perspective, platelet activation during viral infection can be triggered through multiple, often overlapping pathways. These include the recognition of viral components through platelet pattern-recognition receptors, binding of immune complexes with subsequent Fc receptor engagement, complement activation, and indirect stimulation mediated by inflammatory cytokines, neutrophil extracellular traps (NETs), endothelial injury, and thrombin generation [2,3]. Importantly, experimental evidence has demonstrated that platelet innate immune signaling pathways actively contribute to host defense and disease modulation. In murine models of viral infection, activation of platelet Toll-like receptor 7 (TLR7) promotes platelet-neutrophil interactions and significantly influences both platelet count and host survival, supporting the concept that platelets are not passive victims of infection but active participants in viral pathophysiology [1].

The COVID-19 pandemic has further consolidated the concept of platelet-driven immune-thrombosis in viral disease. Translational studies have shown that platelets from patients with SARS-CoV-2 infection exhibit a hyperactivated phenotype and can associate with viral RNA; however, whether this reflects true viral internalization or merely surface binding or uptake of viral fragments remains controversial. Importantly, the presence of viral RNA alone does not constitute definitive evidence of productive infection of platelets, and the expression of ACE2 on platelets has not been consistently confirmed across studies [4]. In parallel, experimental data indicate that the SARS-CoV-2 spike protein can directly activate platelets and enhance platelet-monocyte crosstalk, providing a mechanistic link between hypercoagulability and amplified inflammatory responses [5,6]. Platelets have also been shown to exacerbate endothelial dysfunction in COVID-19, promoting a prothrombotic endotheliopathy and sustaining a self-reinforcing loop between vascular injury and coagulation activation [5]. Moreover, inflammatory and hematopoietic mediators such as thrombopoietin (THPO) are elevated in patients with COVID-19 and may further prime platelet activation and platelet–leukocyte interactions, with potential implications for risk stratification and prognosis [7].

Beyond SARS-CoV-2 infection, dengue virus represents a paradigmatic example of platelet–virus interactions. In dengue, platelet activation has been identified as a key determinant of thrombocytopenia severity, with evidence supporting a combined mechanism of activation-induced consumption and immune-mediated clearance [8]. Furthermore, the dengue virus non-structural protein 1 (NS1) has been shown to directly induce platelet activation and aggregation, providing a biological rationale for vascular dysfunction and the hemorrhagic and thrombotic manifestations observed in severe disease [9]. In addition to NS1, other dengue viral proteins have been implicated in platelet dysfunction. The envelope (E) protein can bind platelet surface receptors and promote activation, while the capsid (C) protein and NS3 protease have been associated with platelet apoptosis and immune-mediated clearance, further contributing to thrombocytopenia and vascular leakage.

Another clinically relevant model is human immunodeficiency virus (HIV) infection, in which platelet activation contributes to immune dysregulation and thrombocytopenia. The HIV-1 Tat protein has been demonstrated to induce platelet activation and CD154 release, potentially promoting autoimmune mechanisms involved in HIV-associated thrombocytopenia [10]. In HIV infection, platelets overexpress several activation markers, including P-selectin, glycoprotein IIb/IIIa, and release increased levels of platelet factor 4 (PF4) and soluble CD40L, which contribute to immune dysregulation and vascular inflammation.

Collectively, these findings support a unifying model in which platelets act as an integrated platform linking immunity, inflammation, and thrombosis during viral infections, thereby critically influencing both viral clearance and host-mediated thrombo-inflammatory injury [2,3,4,5].

## 2. Mechanisms of Platelet–Virus Interactions

Platelets interact with viruses through a complex network of direct and indirect mechanisms that converge on activation, immune modulation, and thrombo-inflammatory amplification. At the surface level, platelets express a wide repertoire of receptors able to bind viral particles or virus-derived ligands, including FcγRIIA, complement receptors, C-type lectins, and pattern-recognition receptors such as Toll-like receptors (TLRs) [11,12,13]. These receptors enable platelets to sense viral nucleic acids and immune complexes, triggering intracellular signaling cascades that promote degranulation, integrin activation, and the release of pro-inflammatory and pro-coagulant mediators.

Among innate immune pathways, TLR7 and TLR9 have emerged as pivotal sensors of viral single-stranded RNA and DNA within platelets. Engagement of these receptors induces platelet activation and promotes interactions with neutrophils and monocytes, thereby linking viral recognition to immune-thrombosis [11,12]. Platelet activation is further amplified by complement deposition on viral particles and immune complexes, which enhances Fc receptor-mediated signaling and accelerates platelet consumption and clearance [13]. In parallel, activated platelets release a broad array of mediators including platelet factor 4 (PF4), RANTES, and CD40L that shape leukocyte recruitment, endothelial activation, and adaptive immune responses [14].

**Figure 2:**
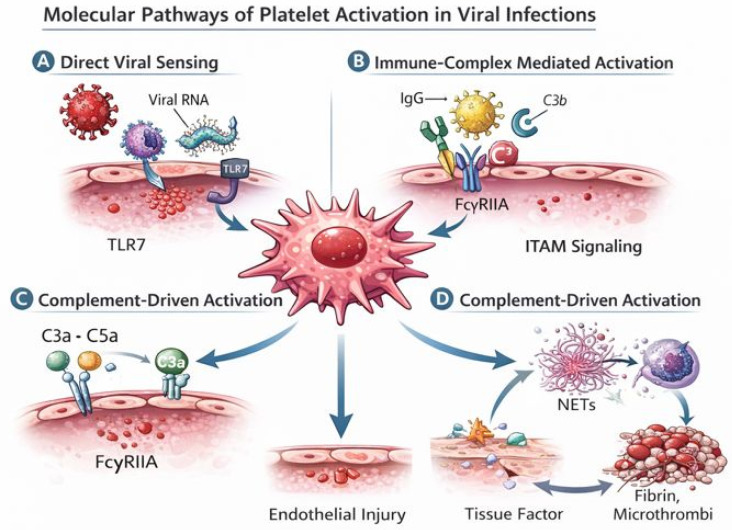
Molecular pathways of platelet activation in viral infections. This figure illustrates the main molecular mechanisms through which viral infections induce platelet activation and drive thrombo-inflammatory responses. (**A**) Direct viral sensing: viral ssRNA associated with platelets (through binding or uptake of viral material) is recognized by Toll-like receptor 7 (TLR7), triggering platelet activation and degranulation. (**B**) Immune complex–mediated activation: virus–IgG complexes bind to the platelet FcγRIIA receptor, leading to ITAM-dependent signaling and amplification of platelet activation. (**C**) Complement-driven activation: complement fragments such as C3a and C5a engage platelet receptors and enhance platelet priming and responsiveness, further promoting inflammatory and procoagulant activity. (**D**) Thrombo-inflammatory amplification loop: activated platelets interact with neutrophils to promote the release of neutrophil extracellular traps (NETs) and the expression of tissue factor, leading to thrombin generation, fibrin deposition, and microthrombus formation, which in turn exacerbate endothelial injury and sustain platelet activation. Together, these interconnected pathways explain how viral sensing by platelets is translated into immune-thrombosis and vascular damage.

While direct viral internalization by platelets has been demonstrated for some viral families (e.g., influenza virus and HIV), evidence for true SARS-CoV-2 internalization remains inconclusive. Observations of viral RNA in platelets have not been consistently replicated, and may reflect surface binding or uptake of viral debris rather than productive infection [15,16,17]. Viral uptake can induce platelet apoptosis-like changes and microparticle release, contributing to thrombocytopenia and to the dissemination of procoagulant phospholipid surfaces [16]. Furthermore, activated platelets readily form aggregates with neutrophils and monocytes, promoting NET formation, tissue factor expression, and thrombin generation, hallmarks of virus-induced immune-thrombosis [14,18]. Collectively, these mechanisms position platelets as central hubs where viral sensing, innate immunity, and coagulation converge. Viral-induced platelet activation is mediated by multiple converging molecular pathways, including direct viral sensing, immune complex signaling, and complement activation, which together amplify thrombo-inflammatory responses (Figure 2). Beyond their role in hemostasis, platelets exert a broad range of immune functions during viral infection, including pathogen sensing, leukocyte recruitment, and regulation of thrombo-inflammatory responses.(Table 1)

Emerging evidence suggests that viral enzymes, particularly viral proteases, may directly modulate platelet activation and survival. Viral proteases such as dengue NS3, HIV protease, and SARS-CoV-2 main protease (Mpro) have been shown to cleave host signaling and cytoskeletal proteins, potentially altering platelet responsiveness, promoting apoptosis-like changes, and enhancing extracellular vesicle release. These viral enzymes may therefore represent an underappreciated mechanistic link between viral replication and platelet-driven immunothrombosis.

## 3. HCV and the Platelet Paradox

Chronic hepatitis C virus (HCV) infection provides a paradigmatic example of the “platelet paradox”, in which thrombocytopenia coexists with a prothrombotic phenotype and clinically relevant thrombotic events [19]. It is essential to distinguish between primary hemostasis, which is primarily governed by platelet number and function, and secondary hemostasis (coagulation), which is largely regulated by liver-derived clotting and fibrinolytic factors. Clinically significant impairment of primary hemostasis typically occurs only when platelet counts fall below 20 × 10^3^/μL. Therefore, the increased thrombotic risk observed in chronic HCV infection cannot be attributed to platelet count per se, but rather reflects profound alterations in the coagulation system driven by chronic liver disease. Endothelial infection and damage, together with chronic inflammation, lead to increased expression of tissue factor (TF) and anti-fibrinolytic mediators such as plasminogen activator inhibitor-1 (PAI-1). Moreover, disruption of the hepatic synthesis of both procoagulant and anticoagulant factors results in a rebalanced but unstable hemostatic system, predisposing to thrombosis despite thrombocytopenia. In chronic HCV, reduced platelet count is common and multifactorial, reflecting hypersplenism and portal hypertension, reduced hepatic thrombopoietin synthesis, immune-mediated platelet destruction, and impaired megakaryopoiesis [20,21]. However, despite this quantitative platelet defect, HCV infection is consistently associated with systemic hypercoagulability and an increased risk of venous thromboembolism (VTE), including deep vein thrombosis and pulmonary embolism [22,23,24]. At the biological level, chronic viral persistence promotes endothelial dysfunction and sustained platelet activation, as reflected by elevated circulating markers such as soluble P-selectin, which correlate with viral replication and inflammatory activity in HCV patients [19]. Moreover, viral hepatitis is strongly associated with portal vein thrombosis, particularly in advanced chronic liver disease, confirming that thrombocytopenia does not confer protection against thrombosis in this setting [25]. Emerging evidence also indicates that extracellular vesicles (EVs) participate in this prothrombotic milieu: plasma EVs isolated from HCV-infected patients display endothelial-damaging and pro-inflammatory properties, providing a mechanistic link between chronic viral infection, vascular injury, and immunothrombosis [26]. Collectively, HCV infection extends the platelet–virus axis beyond acute viral syndromes, demonstrating that numerical platelet depletion can coexist with qualitative platelet hyperreactivity and sustained thrombo-inflammatory risk [19,20,21,22,23,24,25,26]. It is important to note that not all viral hepatitides display the same platelet–coagulation phenotype. For instance, hepatitis A and E, which do not cause chronic liver disease, are not typically associated with sustained platelet activation or systemic hypercoagulability, highlighting the unique pathophysiological role of chronic HCV infection.

## 4. Clinical Implications and Therapeutic Perspectives

Clinically, platelet dysregulation has profound implications across a wide spectrum of viral diseases. Thrombocytopenia, platelet hyperreactivity, and increased platelet–leukocyte aggregates have all been associated with disease severity and adverse outcomes in viral infections such as COVID-19, influenza, dengue, and HIV [16,17,18,19,20,21,22,23,24,25,26,27]. A wide spectrum of clinically relevant viruses is associated with distinct patterns of platelet abnormalities and clinical complications, as summarized in Table 2. Importantly, platelet activation markers, including soluble P-selectin, CD40L, and platelet-derived extracellular vesicles, have emerged as potential biomarkers of thrombo-inflammatory burden and prognosis in viral illnesses [16,18]. These observations support the integration of platelet-related parameters into risk stratification models for patients with severe viral infections [27].

From a therapeutic standpoint, the platelet–virus axis represents a promising but still largely unexplored target. Antiplatelet agents, including aspirin and P2Y12 inhibitors, have been proposed as modulators of virus-induced immuno-thrombosis, with observational data in COVID-19 suggesting potential benefits in reducing thrombotic complications and mortality [28]. At the same time, therapies targeting platelet–leukocyte interactions or platelet innate immune pathways, such as inhibition of TLR-mediated signaling or blockade of CD40L, may offer more selective strategies to attenuate harmful inflammation without compromising hemostasis [11,14,29].

Ebola virus is uniquely characterized by a fulminant consumptive coagulopathy driven by profound endothelial injury, cytokine storm, and massive platelet consumption, resulting in a combined hemorrhagic and thrombotic phenotype that distinguishes it from most other viral infections.

Another emerging avenue involves modulation of complement-platelet crosstalk and viral immune complexes, which contribute to platelet activation and consumption in several viral diseases [13,17]. However, any therapeutic intervention targeting platelets in viral infection must carefully balance the reduction in thrombo-inflammation against the risk of bleeding, particularly in conditions characterized by profound thrombocytopenia such as dengue or viral hemorrhagic fevers [18]. Overall, a deeper mechanistic understanding of the platelet–virus axis will be essential to guide the rational development of antithrombotic and immunomodulatory therapies in viral disease. A summary of the main platelet-related biomarkers implicated in viral diseases, together with their pathophysiological role and clinical relevance, is provided in Table 3.

## 5. Discussion

The emerging concept of the platelet–virus axis provides a unifying framework to interpret why many viral infections display a thrombo-inflammatory phenotype despite variable degrees of thrombocytopenia. Across clinically relevant viral diseases, platelets operate as rapid immune sensors and amplifiers of vascular inflammation, integrating pathogen-derived signals with endothelial injury and coagulation activation (Figure 1). In COVID-19, converging clinical and experimental data support the idea that platelet hyperreactivity and platelet-derived extracellular vesicles (EVs) contribute to immune-thrombosis, partly by providing procoagulant phospholipid surfaces and by sustaining platelet–leukocyte cross-talk [30,31]. More broadly, platelet–leukocyte aggregates appear to be a recurring hallmark of viral immunopathology (including influenza, dengue, HIV and SARS-CoV-2), linking innate immune activation to tissue factor induction, NET formation, thrombin generation, and microvascular thrombosis [32].

A key unresolved issue is whether platelet perturbations in viral infections are predominantly adaptive (host-protective) or maladaptive (driver of organ damage). Evidence increasingly suggests a dual role: platelet innate immune signaling may assist in antiviral responses, yet excessive activation can fuel endothelial dysfunction, thrombin burst, and organ injury [31,32,33]. Recent mechanistic studies reinforce the notion that the inflammatory milieu of severe viral disease contains transferable prothrombotic triggers that prime platelets and promote platelet-dependent Extracellular Vesicles (EV) release and thrombosis; extracellular histones, for example, have been proposed as a “common pathway” coupling inflammation to platelet-driven thrombosis and EV generation in COVID-19 [34]. In parallel, detailed phenotyping of circulating EVs indicates that platelet–leukocyte interaction can be captured at the EV level, with platelet-associated EV aggregates co-expressing leukocyte markers and tissue factor supporting EV-based readouts as candidate biomarkers of immune-thrombotic burden [35]. Collectively, these observations strengthen the rationale for incorporating platelet activation and EV signatures into risk stratification models, particularly in patients with severe viral disease or high thrombotic risk [30,35].

From a therapeutic perspective, the platelet–virus axis remains an attractive but challenging target. Trials aimed at disrupting platelet adhesion pathways have yielded mixed results. P-selectin blockade with crizanlizumab did not improve organ support-free days in hospitalized COVID-19 patients in a randomized controlled trial [36], suggesting that single-pathway inhibition may be insufficient once systemic thrombo-inflammation is established. Similarly, randomized evidence evaluating antiplatelet intensification on top of anticoagulation showed no improvement in organ support-free days among non-critically ill COVID-19 patients receiving therapeutic heparin plus a P2Y12 inhibitor compared with heparin alone, with a numerical increase in major bleeding [37]. In critically ill COVID-19 patients, P2Y12 inhibition also failed to increase organ support-free survival [38]. Taken together, these data indicate that “blanket” antiplatelet strategies may not translate into meaningful clinical benefit in unselected viral pneumonia cohorts and underscore the need for precision approaches, including careful timing, patient phenotyping, and endpoint selection.

A plausible way forward is to align interventions with the dominant biological process in each patient subgroup: (i) targeting platelet–leukocyte interactions and platelet-derived EV generation in patients with clear immune-thrombotic signatures [30,31,35]; (ii) considering upstream inflammatory mediators (e.g., histone-driven platelet activation) as alternative or complementary targets in selected severe cases [34]; and (iii) integrating platelet/EV biomarkers into adaptive trial designs to enrich for patients most likely to benefit [30,35]. Finally, the expanding literature on platelet EVs in viral disease-including both infection- and vaccine-associated contexts-highlights EVs as potential biomarkers and mechanistic effectors and supports their inclusion in future translational pipelines [39]. Overall, the next phase of research should move from descriptive associations to biomarker-guided and mechanism-informed interventions, leveraging platelet-centered phenotyping to refine risk stratification and therapeutic targeting.

## 6. Conclusions

The evidence reviewed in this article supports a paradigm shift in the understanding of viral diseases, positioning platelets as central orchestrators of the host response to infection rather than passive bystanders of systemic inflammation and coagulopathy. Across a broad spectrum of clinically relevant viruses, including SARS-CoV-2, dengue virus, HIV, and influenza, platelets actively sense viral components, interact with immune complexes and complement, and engage in dynamic cross-talk with leukocytes and endothelial cells. Through these mechanisms, platelets integrate innate immune signaling with coagulation and vascular homeostasis, generating the thrombo-inflammatory phenotype that characterizes severe viral illness.

The concept of a platelet–virus axis provides a unifying biological framework to explain the paradoxical coexistence of thrombocytopenia and hypercoagulability observed in many viral infections. Platelet activation, platelet–leukocyte aggregation, extracellular vesicle release, and endothelial priming collectively drive immune-thrombosis, microvascular injury, and organ dysfunction. At the same time, platelet innate immune functions may contribute to antiviral defense, highlighting the dual protective and pathogenic roles of platelets during infection.

From a clinical perspective, platelet-related biomarkers and platelet-derived extracellular vesicles hold promise as tools for risk stratification, disease monitoring, and therapeutic guidance in viral diseases. However, interventional studies targeting platelet pathways in COVID-19 have underscored the complexity of this axis, indicating that indiscriminate antiplatelet therapy is unlikely to be beneficial in unselected patients and may increase bleeding risk. Future strategies should therefore focus on precision medicine approaches, integrating platelet phenotyping, inflammatory profiling, and timing of intervention to identify patients most likely to benefit from modulation of platelet-driven thrombo-inflammation.

Ultimately, a deeper mechanistic understanding of the platelet–virus axis will be essential to translate these insights into effective therapies. By bridging virology, immunology, and thrombosis, this field offers a powerful opportunity to redefine how viral diseases are diagnosed, stratified, and treated, with the potential to substantially improve outcomes in patients affected by severe viral infections.

## Figures and Tables

**Table 1:** Platelet immune functions in viral infection. This table outlines the major immune functions of platelets in the context of viral infections. Platelets are shown to act as immune sentinels through the expression of pattern-recognition receptors, to regulate leukocyte recruitment and activation via surface molecules and soluble mediators, and to promote thrombo-inflammatory responses through the release of extracellular vesicles and the induction of neutrophil extracellular traps (NETs). Together, these functions position platelets as key modulators of both antiviral immunity and immunopathology.

Platelet Function	Role
TLR7/TLR9 sensing	Viral RNA detection
CD40L expression	T-cell activation
PF4, RANTES	Leukocyte recruitment
Microparticle release	Coagulation amplification
NET induction	Immunothrombosis

**Table 2:** Viral infections associated with platelet disorders. This table summarizes the main viral infections associated with platelet abnormalities, highlighting the predominant platelet phenotypes, the underlying pathophysiological mechanisms, and their major clinical consequences. For each virus, alterations in platelet count and function, including activation, immune-mediated clearance, and consumptive coagulopathy, are reported together with the key molecular pathways involved, such as viral protein-platelet interactions, immune complex formation, and endothelial injury. The table illustrates how diverse viral pathogens converge on common platelet-driven mechanisms that contribute to thrombocytopenia, immune-thrombosis, bleeding, and organ dysfunction.

Virus	Platelet Abnormality	Mechanism	Clinical Consequence
SARS-CoV-2	Hyperactivation, thrombocytopenia	Spike–platelet, TLR7	Thrombosis, ARDS
Dengue	Severe thrombocytopenia	NS1, immune clearance	Bleeding, shock
HIV	Immune thrombocytopenia	Tat, CD154	Bleeding, thrombosis
Influenza	Platelet activation	TLR7	Lung microthrombosis
Ebola	Consumptive coagulopathy	Endothelial injury	DIC, bleeding

**Table 3:** Platelet-related biomarkers in viral diseases. This table summarizes the main platelet-derived or platelet-associated biomarkers that have been implicated in the pathogenesis of viral infections. For each biomarker, the predominant viral settings, the underlying pathophysiological mechanisms (including platelet activation, immune modulation, endothelial dysfunction, and coagulation amplification), and the associated clinical implications are reported. These markers reflect the central role of platelets in linking innate immunity with thrombo-inflammatory responses and highlight their potential value for risk stratification, disease severity assessment, and therapeutic monitoring in patients with viral diseases.

Biomarker	Virus	Mechanism	Clinical Relevance
Soluble P-selectin	COVID-19, HCV	Platelet/endothelial activation	Severity, thrombosis risk
CD40L	HIV, COVID-19	Immune activation	Immune dysregulation
PF4	Dengue, HIV	Immune complex formation	Thrombocytopenia
Platelet-derived EVs	COVID-19, HCV	Procoagulant surfaces	Immunothrombosis
Platelet–leukocyte aggregates	Influenza, COVID-19	NETs, TF induction	Microthrombosis

## Data Availability

No new data were created or analyzed in this study.
